# Physical pain – suicidality association in adults: A meta-analysis

**DOI:** 10.1192/j.eurpsy.2021.1549

**Published:** 2021-08-13

**Authors:** M. Rignanese, M. De Filippi, E. Salmè, F. Madeddu, R. Calati

**Affiliations:** 1 Psychology, University of Milano-Bicocca, Milan, Italy; 2 Psychiatry, Nîmes University Hospital, Nîmes, France

**Keywords:** Suicidal Thoughts, suicidal behaviours, meta-analysis, Physical Pain

## Abstract

**Introduction:**

Multiple epidemiologic and clinical studies have explored the relationship between physical pain and suicidal thoughts and behaviours.

**Objectives:**

The aim of this meta-analysis was to provide an update of the data already present in literature about this specific association in adults.

**Methods:**

Starting from a meta-analysis published by Calati and colleagues in 2015, 28 studies were included in this work. After searching on Pubmed (until March 2020), data were extracted from articles comparing the rates of current and lifetime suicidal thoughts and behaviours (death wish, suicidal ideation, suicidal planning, suicide attempt and suicide death: DW, SI, SP, SA, and SD) in adults with any type of physical pain and in individuals who did not report this condition. Data were analysed using Cochrane Collaboration Review Manager software (RevMan, version 5.4).

**Results:**

Although high between-study heterogeneity was detected in most analyses, results suggested that adults with physical pain are more likely to report any form of suicidal outcome, except for death by suicide, compared to individuals not affected by pain. No evidence of publication bias was reported in the main analysis (lifetime SA).

**Conclusions:**

Collected data are therefore in line with previous literature on this topic, which considered physical pain an extremely relevant risk factor for suicidal thoughts and behaviours. Future studies should specifically focus on alternative types of physical pain (such as medically unexplained pain or psychogenic pain) or explore the different impact of acute versus chronic pain in terms of increased suicide risk.

